# Reference genes for QRT-PCR tested under various stress conditions in *Folsomia candida *and *Orchesella cincta *(Insecta, Collembola)

**DOI:** 10.1186/1471-2199-10-54

**Published:** 2009-06-01

**Authors:** Muriel E de Boer, Tjalf E de Boer, Janine Mariën, Martijn JTN Timmermans, Benjamin Nota, Nico M van Straalen, Jacintha Ellers, Dick Roelofs

**Affiliations:** 1 Institute of Ecological Science, VU University Amsterdam, De Boelelaan 1085, 1081 HV, Amsterdam, The Netherlands; 2 Current address: Faculty of Natural Sciences, Department of Ecology & Evolution, Imperial College London Exhibition Road, London SW7 2AZ, UK

## Abstract

**Background:**

Genomic studies measuring transcriptional responses to changing environments and stress currently make their way into the field of evolutionary ecology and ecotoxicology. To investigate a small to medium number of genes or to confirm large scale microarray studies, Quantitative Reverse Transcriptase PCR (QRT-PCR) can achieve high accuracy of quantification when key standards, such as normalization, are carefully set. In this study, we validated potential reference genes for their use as endogenous controls under different chemical and physical stresses in two species of soil-living Collembola, *Folsomia candida *and *Orchesella cincta*. Treatments for *F. candida *were cadmium exposure, phenanthrene exposure, desiccation, heat shock and pH stress, and for *O. cincta *cadmium, desiccation, heat shock and starvation.

**Results:**

Eight potential reference genes for *F. candida *and seven for *O. cincta *were ranked by their stability per stress factor using the programs geNorm and Normfinder. For *F. candida *the succinate dehydrogenase (*SDHA*) and eukaryotic transcription initiation factor 1A (*ETIF*) genes were found the most stable over the different treatments, while for *O. cincta*, the beta actin (*ACTb*) and tyrosine 3-monooxygenase (*YWHAZ*) genes were the most stable.

**Conclusion:**

We present a panel of reference genes for two emerging ecological genomic model species tested under a variety of treatments. Within each species, different treatments resulted in differences in the top stable reference genes. Moreover, the two species differed in suitable reference genes even when exposed to similar stresses. This might be attributed to dissimilarity of physiology. It is vital to rigorously test a panel of reference genes for each species and treatment, in advance of relative quantification of QRT-PCR gene expression measurements.

## Background

Genomic techniques have undergone major developments in the last two decades. As a result, they have become conducive for evolutionary and ecotoxicological studies, which generally use non genomic-model organisms. Quantitative Reverse-Transcriptase Polymerase Chain Reaction (QRT-PCR) is a technique to estimate gene expression levels. This technique is often used to confirm high throughput systems like microarrays. Its application has mainly been limited to small numbers of genes per experiment due to constraints of low throughput coinciding with relatively high costs per assay. This is about to change, as high throughput QRT-PCR systems using small volume (capillary) PCR are becoming available [1]. QRT-PCR is a valuable tool for ecological studies as it provides a relatively straightforward way to measure the direct transcriptional response of an organism exposed to different treatments [2]. QRT-PCR has been applied to study adaptive evolution at the transcriptional level [3-6]. For instance, Roelofs et al. [7] conducted a QRT-PCR study to assess the relevance of transcriptional regulation in the adaptive evolution of stress tolerance.

The conditions that have to be met for a successful QRT-PCR experiment are reviewed by Bustin [8]. Different strategies have been developed for quantifying gene expression with QRT-PCR data. The most widely used method is to quantify the relative amount of target mRNA between samples, using for instance the comparative CT (2^-ΔΔCT^) method [9], or the more comprehensive Pfaffl method where relative quantities are adjusted for amplification efficiencies. Relative quantification methods depend on reference genes for normalization [10]. QRT-PCR reference genes, sometimes called 'housekeeping' genes, can either be internal, when measured in the same reaction tube as the target gene, or external, when measured in a different reaction tube. The use of reference genes is necessary to correct for factors such as RNA input differences and reverse transcriptase efficiency variation [11].

An essential requirement for QRT-PCR reference genes is stability of their transcriptional level across the various conditions to which an organism is exposed during an experiment. Classically, the most used reference genes are carry-overs from the Northern-blot days [12] such as beta actin (*ACTb*), glyceraldehyde-3P-dehydrogenase (*GAPDH*) and ribosomal RNA genes (*18S*, *28S*). Unfortunately, these classical reference genes are not always found to be suitable for this use. For example, mRNA levels of *ACTb *and *GAPDH *can fluctuate widely in human T-cells exposed to different treatments [13]. Also, commonly used housekeeping genes like *ACTb*, *GAPDH*, cyclophilin A (*CYP*) and *28S *are up or down regulated in cell lines exposed to hypoxia stress [14]. Therefore, it is vital to validate the stability of a panel of reference genes in order to select the most suitable ones for each new treatment and species of choice.

Currently two Visual Basic Applications for Microsoft Excel are widely used to determine reference gene suitability: geNorm [15] and Normfinder [16]. GeNorm is based on the principle that the expression ratio of two ideal control genes should be identical in all samples and experimental conditions. It calculates gene expression stability (M), which is the mean pair-wise variation between an individual gene and all other tested reference genes. Subsequently, the least stable reference gene (highest M value) is excluded from the set and the calculation is reiterated until the two most stable reference genes remain. Normfinder [16] estimates intergroup and intragroup variation to calculate reference gene stability and to rank them. As with geNorm it calculates a stability value for each potential reference gene but it uses a variance model based approach (mixed linear effect modeling), instead of the reiterative approach used by geNorm. Normfinder calculates intragroup variability for the genes in each of the groups, and the intergroup variability or bias among the groups [17]. Since the program can differentiate between groups, Normfinder is best suited when the stability of reference genes needs to be assessed over multiple treatments. When reference gene stability only needs to be calculated for samples exposed to a single treatment the two methods are similar and should give the same results [16].

In this paper we develop a panel of reference genes for two species of springtails (Collembola), and study their stability across various treatments. Collembola are important model organisms in evolutionary ecology and ecotoxicological studies. The soil-dwelling collembolan *Folsomia candida *is a standard test animal used in a soil pollution test that is certified by the International Organization for Standardization [18]. Its parthenogenetic mode of reproduction and the availability of a recently sequenced Expressed Sequence Tag (EST) database, make *F. candida *also a proper test animal for genomic studies on the effects of soil pollution [19-21]. An *F. candida *microarray is currently in use for testing soil toxicants [22] and physical conditions (Timmermans et al., unpubl. data; De Boer et al., unpubl. data). Therefore, confirmation of microarray results by QRT-PCR is abound. *Orchesella cincta *is a sexually reproducing collembolan that lives in the litter layer rather than in the soil and is generally used to study adaptation and phenotypic plasticity [23,24]. Furthermore, it is an emerging genomic model to study adaptive evolution in polluted environments [20,21,25].

Here we study the stability of potential QRT-PCR reference genes across various treatments. We exposed the two collembolan species to several stressors (*F. candida*: cadmium, phenanthrene, desiccation, temperature and pH stress. *O. cincta*: cadmium, desiccation, temperature stress and starvation) that are currently under investigation in gene expression studies. Based on previous studies (e.g. [12,26]), a panel of potential QRT-PCR reference genes was developed (Table 1) using the Collembola EST database Collembase [20]. In addition, a target gene (expected to be differential) was measured for each treatment, to validate the impact of the treatment at the transcriptional level of the organism. The stability of the potential reference genes was determined using both geNorm and Normfinder.

**Table 1:** Overview of reference genes and differentially expressed genes in *Folsomia candida *and *Orchesella cincta*

**Gene name**	**Symbol**	**GenBank Accesion no.**	**Collembase id**	**Gene Ontology**
Beta Actin	*ACTb*	FC: EV473840 OC: AY779737	Fcc01756 n/a	structural constituent of cytoskeleton (F) GO:0005200
Glyceraldehyde 3-Phosphatase dehydrogenase	*GAPDH*	FC: EV479869 OC: FJ009068	Fcc05545 n/a	Glycolysis (P) GO:0006096
Ubiquitin conjugating enzyme	*UBC*	FC: EV475860 OC: n/a	Fcc00615 n/a	Ubiquitin-protein ligase activity (F) GO:0004842
Succinate dehydrogenase	*SDHA*	FC: EV476739 OC: FJ009079	Fcc06005 n/a	Tricarboxylic acid cycle (P) GO:0006099
Tyrosine 3-monooxygenase	*YWHAZ*	FC: EV474941 OC: n/a	Fcc02512 Occ00412	Protein domain specific binding (F) GO:0019904
Elongation factor 1-alpha	*EF1a*	FC: EV473706 OC: AH009877	Fcc05454 n/a	Protein biosynthesis (P) GO:0006412
Eukaryotic Transcription Initiation Factor 5a	*ETIF*	FC: EV479461 OC: n/a	Fcc02111 n/a	Protein biosynthesis (P) GO:0006412
Cyclophilin A	*CYP*	FC: EV475615 OC: n/a	Fcc01655 n/a	Protein folding (P) GO:0006457
28S	28*S*	FC: n/a OC: AF483443	n/a n/a	Large ribosomal subunit (C) GO0022625
Alpha Tubulin	*TBa*	FC: n/a OC: GD180623	n/a n/a	contributes to the structural integrity of a cytoskeletal structure (F) GO:0005200
Heat Shock Protein 70	*HSP70*	FC: EV473626 OC: FJ009069	Fcc01609 n/a	Response to unfolded protein (P) GO:0006986
V-type ATPase	*ATPase*	FC: EV476428 OC: n/a	Fcc04630 n/a	Hydrogen transport activity (F) GO:0015078
Cuticle Protein	*CP*	FC: EV479600 OC: n/a	Fcc04701 n/a	a molecule that contributes to the structural integrity of a cuticle (F) GO:0042302
Mitochondrial chaperone BCS1	*BCS1*	FC: EV473062 OC: n/a	Fcc00101 n/a	ATP binding (F) GO:0005524
Metallothionein	*MT*	FC: n/a OC: AF036345	n/a Occ00204	Metal ion binding (F) GO:0046872

## Results

### Expression levels under different conditions

**Figure 1:**
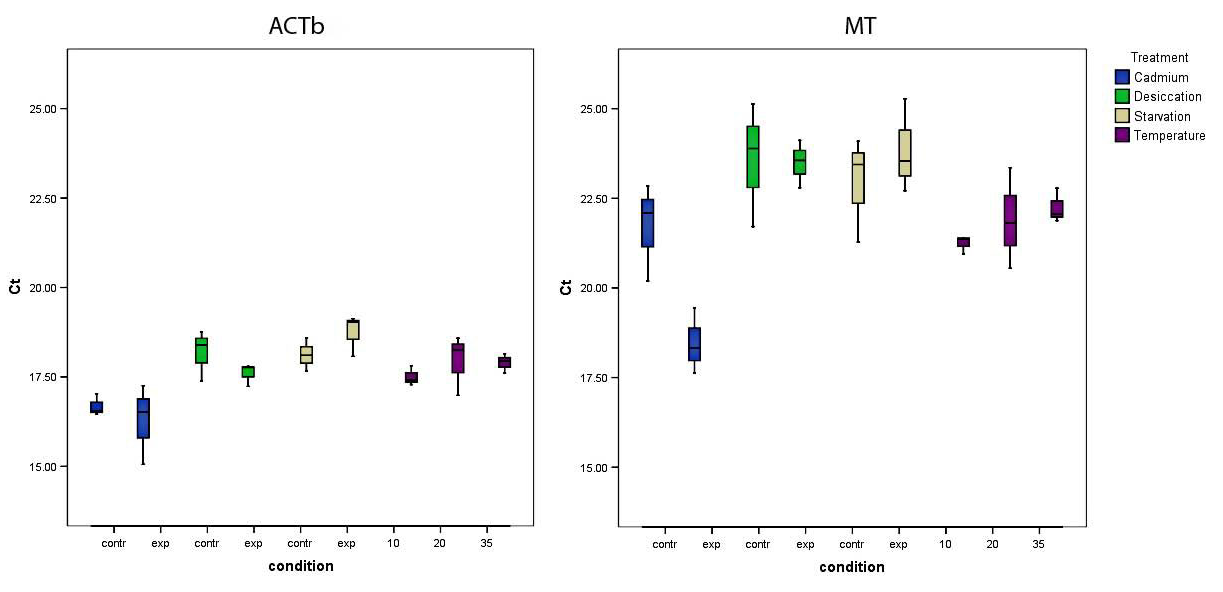
**Ct values of 2 genes from *Orchesella cincta *exposed to the different treatments**. Average Ct values between the biological replicates of the beta actin (ACTb) and metallothionein (MT) genes from *Orchesella cincta *exposed to the cadmium, desiccation, starvation and temperature treatments. Exp stands for the exposed group, contr for the control group. 0, 10 and 35 in the temperature treatment stand for the different temperatures (in °C) the animals were exposed to.

Average cycle threshold (Ct) values varied widely between conditions and treatments, and ranged between 9.7 (*28S, O. cincta*) and 28.6 (*CYP, F. candida*, see Additional file 1 for an overview). An example of the different expression levels in treatments and conditions is given in Figure 1 for *ACTb *and the differential metallothionein gene (*MT*) of *O. cincta*.

In *O. cincta*, the target gene *MT *showed a 7 fold up regulation (FR) in the cadmium treatment (P < 0.05) and *HSP70 *was 5 fold up regulated after heat shock (P < 0.001). Starvation and desiccation treatments showed no significant changes in either of the two target genes, implying that those treatments had fairly small effects, at least on the transcriptional response of these two genes. In *F. candida*, all but one treatment showed a significant effect on the target genes (cadmium: mitochondrial chaperone BCS-1 (*BCS1*), FR = 20, P < 0.0001; phenanthrene: *BCS1*, FR = 6.4, P = 0.001; temperature: *HSP70*, FR > 2, P < 0.001 (for all conditions); desiccation: Cuticle protein (*CP*), FR = 4, P < 0.05). The pH treatment showed no significant differences in the target V-type ATPase (*ATPase*); in fact the *ATPase *gene used in the pH treatment was more stable than most reference genes, again implying that this treatment had only a modest effect on the transcriptional level of this gene.

### Ranking of the reference genes

Stability rankings were established using geNorm and Normfinder. As described in the methods section, the Normfinder analysis should be executed with genes that show no substantial treatment-specific response [17]. Therefore, differential genes were excluded in most cases as well as some of the other genes that showed a systematic response to the treatment (see Table 2 for an overview).

**Table 2:** Omitted and remaining genes and their absolute total biases after preselection for Normfinder analysis

***Folsomia candida***						***Orchesella cincta***					
**Treatment**	**Bias treshold**	**Omitted genes**	**Bias**	**Remaining genes**	**Bias**	**Treatment**	**Bias treshold**	**Omitted genes**	**Bias**	**Remaining genes**	**Bias**

Temperature	2.24	*YWHAZ*	3.78	*ETIF*	1.01	Temperature	1.08	*MT*	1.38	*TBa*	0.24
		*HSP70*	4.95	*UBC*	1.05			*HSP70*	3.49	*SDHA*	0.32
				*SDHA*	1.19					*YWHAZ*	0.45
				*EFIa*	1.38					*EF1a*	0.51
				*CYP*	1.42					28*S*	0.58
				*GAPDH*	1.74					*ACTb*	0.71
				*ACTb*	2.02					*GAPDH*	0.87
Desiccation	1.72	*GAPDH*	2.19	*UBC*	0.43	Desiccation	0.16	*SDHA*	0.26	*HSP70*	0.02
		*CP*	4.99	*CYP*	0.70			28*S*	0.27	*GAPDH*	0.06
				*EFIa*	0.70			*MT*	0.34	*YWHAZ*	0.07
				*ETIF*	0.97					*EF1a*	0.09
				*YWHAZ*	1.13					*TBa*	0.10
				*SDHA*	1.18					*ACTb*	0.15
				*ACTb*	1.54						
Cadmium	1.44	*ETIF*	1.64	*ACTb*	0.20	Cadmium	0.76	*GAPDH*	0.81	*HSP70*	0.01
		*BCS1*	4.31	*EFIa*	0.32			*MT*	2.88	*ACTb*	0.03
				*CYP*	0.84					*SDHA*	0.27
				*YWHAZ*	0.90					28*S*	0.29
				*SDHA*	0.95					*YWHAZ*	0.44
				*GAPDH*	1.11					*TBa*	0.50
				*UBC*	1.24					*EF1a*	0.60
Phenanthrene	0.85	*ETIF*	0.89	*UBC*	0.14	Starvation	0.33	*MT*	0.35	*ACTb*	0.07
		*BCS1*	3.00	*ACTb*	0.21			*EF1a*	0.55	*HSP70*	0.09
				*EFIa*	0.33			*GAPDH*	0.58	*YWHAZ*	0.13
				*YWHAZ*	0.37			*SDHA*	0.67	*TBa*	0.14
				*SDHA*	0.38					28*S*	0.16
				*GAPDH*	0.55	All treatments	1.21	*total RNA*	2.06	*ACTb*	0.34
				*CYP*	0.79			*MT*	3.33	*SDHA*	0.53
pH	0.67	*EFIa*	0.67	*ETIF*	0.36					*TBa*	0.55
		*GAPDH*	0.81	*UBC*	0.38					28*S*	0.91
		*YWHAZ*	0.90	*SDHA*	0.49					*YWHAZ*	1.00
		*CYP*	1.02	*ACTb*	0.57					*EF1a*	1.04
				*ATPase*	0.59					*GAPDH*	1.05
										*HSP70*	1.20

Pre-selection of genes prior to Normfinder analysis based on a threshold for maximum allowable bias of 0.13 times the standard deviation of the total absolute bias of a treatment. For each treatment, bias thresholds are given as well as the corresponding omitted and remaining genes and their absolute total biases per treatment (total RNA input was only included in the All treatment analysis for *Orchesella cincta*).

The stability rankings generated by geNorm or Normfinder were largely similar, even though the ranking order of the genes differed to some extent. An overview of which genes to use for each treatment is found in Table 3. From the three treatments tested for both species, only the cadmium treatment gave similar results between *F. candida *(tyrosine 3-monooxygenase (*YWHAZ*), succinate dehydrogenase (*SDHA*) and *GAPDH*) and *O. cincta *(alpha-tubulin (*TBa*) – not tested in *F. candida *– *SDHA *and *YWHAZ*). To compare interspecies parallels, an additional geNorm analysis was done including only the reference genes available for both species (see Additional file 2). This did not change the fact that most rankings did not correspond between species. In *F. candida*, overall analyses show the same outcomes for the cadmium treatment and the phenanthrene treatment: *YWHAZ, SDHA, GAPDH*. For the temperature and desiccation treatments (*F. candida*) results also overlapped with *SDHA ETIF *and elongation factor 1α (*EF1a*) being the best suited reference genes. The results for *O. cincta *seem to be quite variable. The commonly used reference gene *ACTb *is placed in all top rankings, but not in the cadmium treatment. *28S*, a well known but also controversial reference gene to use, showed a high stability in the temperature treatment. Also remarkable is the stability of *HSP70*, expected to be differential, in the desiccation and starvation treatments. This result has been reported previously for *Drosophila melanogaster*, which did not show an increase in *HSP70Aa *mRNA levels in response to starvation and desiccation [27]. Despite the fact that the ranking of reference genes shows no typical overall uniformity over all different treatments, the most generally applicable reference genes are suggested to be: *ACTb*, *YWHAZ *and *TBa *for *O. cincta *and *SDHA*, *ETIF *and *YWHAZ *for *F. candida*.

**Table 3:** Most stable reference genes per treatment calculated by geNorm and Normfinder

***Folsomia candida***					***Orchesella cincta***				
**Treatment**	**Top 3 most stable genes**		**Optimum no. genes**		**Treatment**	**Top 3 most stable genes**		**Optimum no. genes**	

	**Normfinder**	**GeNorm**	**Norm finder**	**GeNorm**		**Norm finder**	**GeNorm**	**Normfinder**	**GeNorm**

Temperature	*SDHA*	*SDHA*	2	>3	Temperature	28*s*	*YWHAZ*	>3	2
	*ETIF*	*ETIF*				*ACTb*	*ACTb*		
	*EF1a*	*EF1a*				*YWHAZ*	28*s*		
Desiccation	*SDHA*	*SDHA*	2	3	Desiccation	*ACTb*	*ACTb*	2	2
	*ETIF*	*ETIF*				*GAPDH*	*GAPDH*		
	*EF1a*	*GAPDH*				*EF1a*	*EF1a*		
Cadmium	*YWHAZ*	*YWHAZ*	3	2	Cadmium	*TBa*	*TBa*	>3	2
	*SDHA*	*SDHA*				*SDHA*	*YWHAZ*		
	*GAPDH*	*GAPDH*				*YWHAZ*	*SDHA*		
Phenanthrene	*SDHA*	*SDHA*	2	2	Phenanthrene	n/a	n/a	n/a	n/a
	*YWHAZ*	*YWHAZ*				n/a	n/a		
	*GAPDH*	*GAPDH*				n/a	n/a		
pH	*UBC*	*ACTb*	>3	>3	pH	n/a	n/a	n/a	n/a
	*ETIF*	*CYP*				n/a	n/a		
	*ACTb*	*EF1a*				n/a	n/a		
Starvation	n/a	n/a	n/a	n/a	Starvation	*ACTb*	*TBA*	>3	2
	n/a	n/a				*YWHAZ*	*YWHAZ*		
	n/a	n/a				*TBa*	*SDHA*		
All treatments	n/a	n/a	n/a	n/a	All treatments	*ACTb*	n/a	n/a	n/a
	n/a	n/a				*TBa*	n/a		
	n/a	n/a				*YWHAZ*	n/a		

### Optimum number of reference genes

The optimum numbers of reference genes are shown in Table 3. In only two out of nine treatments the two methods agreed on the number of genes to use for normalization (two genes in these cases). However, in nearly all cases either geNorm or Normfinder recommended the use of two reference genes. Comparing the two sets proposed by either program, the differences in significance levels and relative expression levels of a differential gene were found to be very small.

### Effect of method of normalization on relative expression of HSP70

We selected the temperature treatment with the differential *HSP70 *to illustrate the effects of reference gene selection on the calculated relative expression level of a gene of interest. This treatment was chosen because there were four different conditions and *HSP70 *expression clearly responded differently to each of these conditions.

**Figure 2:**
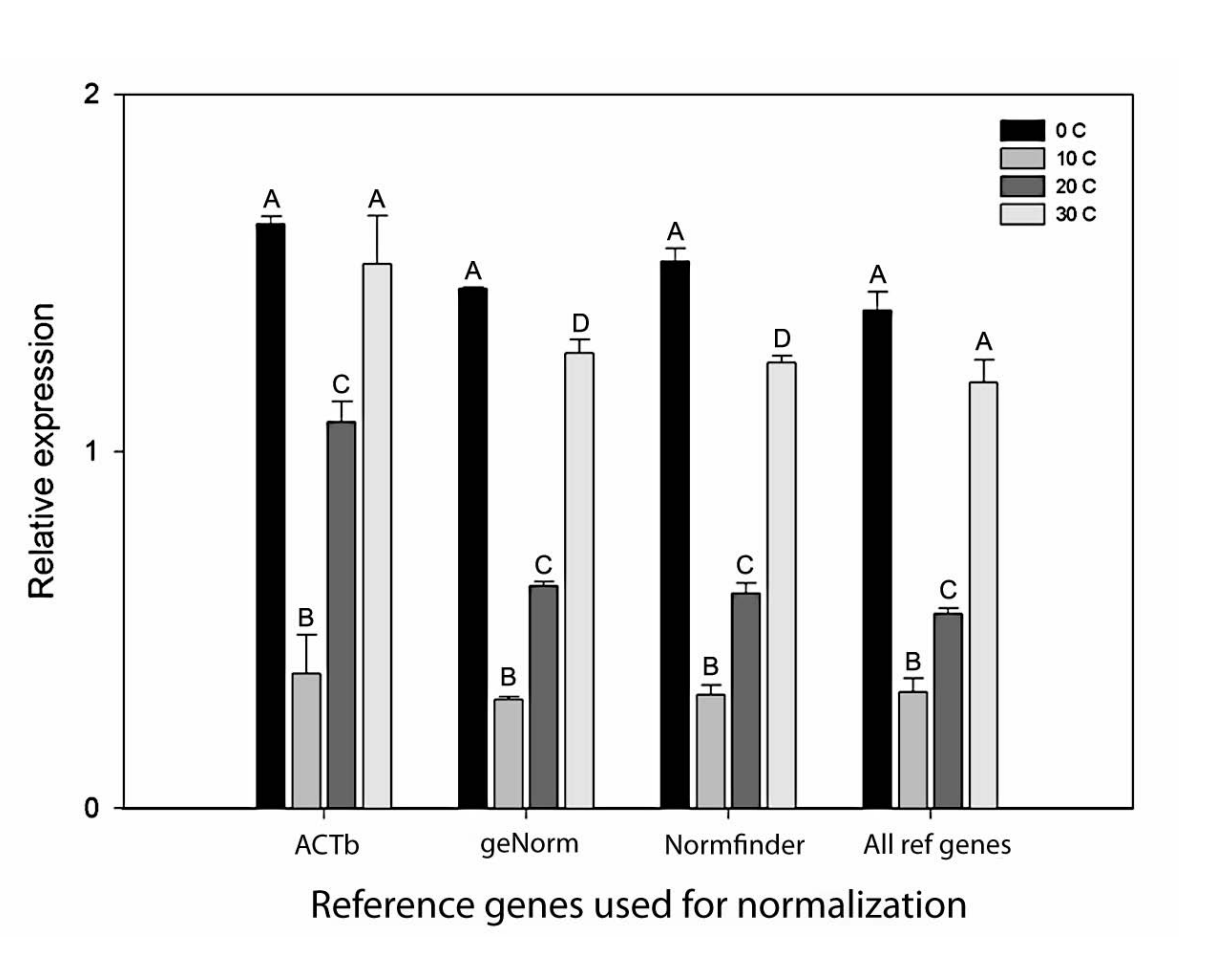
***HSP70 *expression of *Folsomia candida *in the temperature treatment normalized with four different sets of reference genes**. *HSP70 *expression between different pools of *F. candida *exposed to different temperatures, normalized with four different sets of reference genes (ACTb: only beta-actin; the best set selected by geNorm (*SDHA *&*ETIF*); the best set selected by Normfinder (*ETIF*, *SDHA*, *EF1a *&*UBC*) and all available reference genes). One-way ANOVA analysis with Bonferroni post-hoc test revealed significant differences (P < 0.05) between 0, 10 and 20°C and between 10, 20 and 30°C in all normalization sets. Significant differences between 0 and 30°C were only detected when normalized with the optimum sets of reference genes selected by either geNorm or Normfinder. The letters above the bars indicate significant differences where different letters between bars represent a significant difference within 1 normalization set.

We normalized *HSP70 *expression in the *F. candida *temperature treatment with four different sets of reference genes: i) only with a commonly used single reference gene (*ACTb*); ii) with the appropriate number of selected genes according to the geNorm and iii) the Normfinder analysis and iv) with all available reference genes in the *F. candida *temperature dataset. Each set of reference genes showed significant upregulation of *HSP70 *expression in the animals exposed to 0°C and 30°C as compared to those exposed to 10°C and 20°C (P < 0.05), as well as a significant difference between 10°C and 20°C (Figure 2). However, the difference in *HSP70 *expression between 0°C and 30°C was only significant when using the reference genes proposed by the geNorm and Normfinder analysis. This indicates that selection of reference genes can influence the resolution with which differences in gene expression between two samples can be detected.

### Ranking *O. cincta *reference genes over all treatments

Making comparisons between experiments is difficult when no standardization of reaction conditions has taken place [28]. Reverse transcription is the most crucial step in the introduction of technical sampling variation in the QRT-PCR procedure [29]. In the *O. cincta *dataset we attempted to meet the standards needed for a multi-treatment analysis, which allowed us to analyze the whole *O. cincta *dataset similarly to the single treatment analyses using Normfinder. We also included a non-normalized situation ('total RNA input') which was equal for all samples. The differential *MT *was omitted from ranking due to bias beyond the bias threshold as well as 'total RNA input' (Table 2, see materials section and Additional file 3. Although geNorm is less suited to handle heterogeneity than Normfinder, both algorithms produced the same top three of generally applicable reference genes: *ACTb TBa*, and *YWHAZ*.

## Discussion and Conclusion

In this study we identified appropriate reference genes of two collembolan species that show invariant expression across experimental treatments. The most important result of our study is the lack of similarity in stability of genes i) within the species upon exposure to different treatments, and ii) between species that have undergone similar treatments. The two collembolan species have a distinct physiology and ecology, but they belong to phylogenetically related families [30]. An earlier study on two flatfish species with a comparable phylogenetic relatedness as the two collembolan species used here, found congruent results in reference gene stabilities between the two species [31]. In our study, differences were most pronounced in the temperature and desiccation treatments, while the cadmium treatments show a greater congruency between species. It may be argued that the physiological response to xenobiotic stress is more similar in the two species than responses to the environmental factors. Still, experimental methods also differed between species and therefore it cannot be excluded that part of the observed interspecific differences was due to differences in experimental set-up.

Previously, it has been suggested that the results of reference gene selection studies might serve as a resource for future gene expression studies in the same or related species [31,32]. Indeed, some genes were stable across different treatments. *ACTb *and *YWHAZ *were the dominant genes found in the top of the rankings of three out of four *O. cincta *treatments, but this general pattern did not hold up for *ACTb *in the cadmium treatment and *YWHAZ *in the desiccation treatment. In *F. candida*, the two chemical treatments (cadmium and phenanthrene) and the environmental treatments (temperature, desiccation and pH-stress) differed in selected reference genes, indicating that different classes of physiological responses, for instance detoxification and acclimation, can be expected to cause fluctuations in different 'housekeeping' genes to maintain cellular homeostasis. Therefore one cannot assume that two species responding similarly to a certain treatment will also respond in a similar way to another treatment. In addition, differences in priming strategy and QRT-PCR assay characteristics can also introduce variation in stability comparisons. In the light of our present results we must caution against the use of literature data from related species to select a normalization standard, unless careful notion is taken of the parallels and differences in the technical context of the experiment as well as the internal processes of the organisms that are being studied.

Optimizing a set of reference genes not only requires making the right choice of genes, it also implies choosing the right number of genes. The difference in calculated levels of *HSP70 *expression, using different sets of reference genes, exemplifies the importance of the number of reference genes used. Resolution was too low to detect small differences in expression, when either a single reference gene was used for normalization or when all available reference genes were included. In fact the small difference between 0°C and 30°C was only statistically significant when normalization was done with the sets proposed by geNorm and Normfinder.

In the geNorm analysis, inclusion of a differential gene gives insight into the variability of all other genes [15]. Hypothetically, this differential gene should be the most unstable gene in the dataset. In both our species and all treatments (except for the pH treatment) at least one of the selected differential genes was indeed the most variable of the set tested. This provides evidence that the applied treatments caused an effect at the transcriptional level, which strengthens the validation of the reference genes that remained stable under the changed regimes. The only exception was observed for the pH treatment, where the pre-selected differential gene was positioned among the housekeeping genes. Most likely, the treatment may not have been severe enough or it may indicate that some exposure types do not initiate an effect on the transcriptional level.

Normfinder performs relative comparisons between the potential reference genes; hence this method is more sensitive to the presence of differentially expressed genes in the dataset than geNorm. In this study, we set a threshold for the maximal allowable bias at 0.13 times the standard deviation of the treatments' intergroup variation as calculated with Normfinder. After the pre-selection, the reference genes selected by geNorm and Normfinder were remarkably similar. In all treatments, except for the *F. candida *pH treatment and the *O. cincta *starvation treatment, the top three consisted of the same three genes even though the order differed.

QRT-PCR has proven its value in many areas of genetic and genomic research. Knowledge on genetic pathways and molecular responses to external environmental cues and chemical factors now also make a significant contribution to evolutionary ecology and ecotoxicology. Experiments in these scientific disciplines often focus on non-model organisms, such as Collembola, and routinely use large sample sizes. Now that high-throughput systems QRT-PCR are becoming available [1], the technique will become even more valuable for molecular ecological research. The reference genes presented in this study can therefore act as a starting point for scientists who use identical collembolan species for ecological, evolutionary and ecotoxicological research. Nonetheless, as previously stated by Stürzenbaum and Kille [33], it should always be kept in mind that techniqually successful QRT-PCR depends not only on the genes of interest alone, and therefore reference genes should be carefully validated prior to experimentation.

## Methods

### Collembola cultures

*Folsomia candida *was kept in plastic containers with a water-saturated plaster of Paris bottom containing 10% charcoal at 20°C in a 12:12 light dark regime. The animals were fed dried baker's yeast (Dr. Oetker) ad libitum. For all experiments animals of at least 20 days old were used.

*Orchesella cincta *was held comparably, but fed algae (*Desmococcus *sp) growing on twigs of pine trees. For all the experiments animals of 4–5 weeks old were used, with a maximum age difference of seven days.

### *Folsomia candida *treatments

#### pH & temperature treatment

Animals of 20 days old were exposed in 100 ml jars with 30 grams of OECD artificial soil. OECD soil pH was adjusted with CaCO_3 _(J.T. Baker) to four different values (3.5, 4.5, 5.5 & 6.5) according to OECD guideline 207 [34]. Pools of thirty animals per jar were exposed to the different pH values for three days at 75% humidity and 20°C. For the temperature treatment OECD soil at a pH of 5.5 was used. Pools of thirty animals per jar were exposed to 0, 10, 20 and 30°C for 3 days. For each condition two biological replicates were used (separately) for RNA extraction.

#### Desiccation treatment

Drought exposure was performed as described by Bayley and Holmstrup [35]. Per replicate, a pool of twenty-five to thirty animals was exposed in plastic containers to a relative air humidity controlled at 98.2% by placing a NaCl solution of 31.6 g L^-1 ^inside the closed container. Animals were sacrificed after 8, 27, 53 and 174 hours of exposure for RNA isolation. For each condition two biological replicates were used (separately) for RNA extraction.

#### Cadmium and phenanthrene treatment

For the cadmium and phenanthrene treatments animals were exposed in 100 ml jars on a compressed layer of 10 g wet weight of LUFA 2.2 soils (for details see [36]). Procedures of the standard ISO protocol 11267 [18] were followed to spike the soils to nominal concentrations equivalent to the LC50 28 days for cadmium (6.86 mmol kg^-1 ^dry soil [37]) and phenanthrene (422 μmol kg^-1 ^dry soil [36]). For the cadmium spiking a solution of hydrated CdCl_2 _(purity 99%; J.T. Baker, The Netherlands) was used, while phenanthrene (purity 96%; Sigma-Aldrich Chemie, Germany) was dissolved in acetone (Riedel-de Haën, Seelze, Germany), followed by an overnight evaporation time for the acetone. Both soils did not undergo a period of aging. Pools of 15 animals per jar were exposed for a period of 48 hr (cadmium) or 96 hr (phenanthrene) to spiked and clean control soils. For each condition four biological replicates were used (separately) for RNA extraction.

### *O. cincta *treatments

#### Cadmium treatment

Cadmium exposure was performed as described in Roelofs et al. [25]. Animals were exposed individually to a nominal concentration of 1 μmole cadmium per gram dry weight food (algal paste). The exposure was started immediately after moulting to exclude hormonal effects on gene expression and lasted for three days. Five individuals were pooled per replicate; a control was used with the same set-up but fed clean algae. For each condition three biological replicates were used (separately) for RNA extraction.

#### Temperature exposure

Springtails were exposed in glass vials containing slightly moistened foam at the bottom and moistened foam stoppers. Five individuals per vial were exposed to three temperature treatments: cold (10°C), control (20°C) and heat (35°C). Temperature treatments consisted of four hours at 10°C respectively 20°C in a climate room, or one hour placement in a water bath of 35°C with a one hour recovery period at 20°C. For each condition three biological replicates of five pooled animals were used (separately) for RNA extraction.

#### Desiccation treatment

The desiccation treatment followed the protocol described by Bahrndorff et al. [38]. Springtails were exposed to 97.2% relative humidity in a tightly sealed container containing a NaCl solution of 50.66 g L^-1 ^at 20°C for five days. The control treatment followed the same protocol, but instead of the NaCl solution demineralized water was used. We used five individuals per vial and three vials per treatment. Animals from each vial were pooled for RNA isolation.

#### Starvation treatment

We used five individuals per vial and three vials for the starvation treatment and its control. Springtails were transferred to glass vials containing slightly moistened foam at the bottom and moistened foam stoppers. In the control treatment food was made available by adding a piece of bark overgrown with green algae to the animals, while the animals in the starvation treatment did not have access to food. The experimental vials were kept at 20°C in a 12:12 light dark regime for 8 days. Animals from each vial were pooled for RNA isolation.

### RNA isolation and reverse transcription

After exposure animals were snap-frozen in liquid nitrogen and total RNA was isolated with the SV Total RNA isolation system (Promega) according to manufacturer's instructions. Genomic DNA was removed via a DNAse treatment supplied with the kit. RNA integrity was confirmed on a 1% agarose gel and RNA quantities were assessed with a nanodrop ND-1000 spectrophotometer (Nanodrop Technologies) and ranged between 30 and 100 ng μL^-1 ^of total RNA. As indicated by 260/280 and 260/230 nm ratios, all samples used in this study were assumed free from protein contamination and (organic) salts. Absence of amplicons after PCR with Taq-polymerase (MRC Holland, The Netherlands) and 1 μL RNA solution confirmed that no trace DNA contamination was present in the samples used in the further analyses.

Synthesis of cDNA was performed using the reverse-transcriptase system of Promega with the M-MLV reverse transcriptase enzyme and an oligo-dT primer (*F. candida *samples) or random hexamer primers (*O. cincta*). Random hexamer primers were used in the case of *O. cincta *because 28S ribosomal RNA was used as one of the potential reference genes. In the case of the *O. cincta *samples, reverse transcription input amounts were equalized by diluting the total RNA concentrations to 0.5 μg μL^-1^, and samples were reverse transcribed together in a single run. cDNA samples were diluted 4 times before QPCR was carried out. All treatment conditions were reverse transcribed in triplicate, except for the pH treated samples which were performed in duplicate.

### QPCR

Besides reference genes, one or two differentially expressed genes were included in order to observe transcriptional effects of the treatment. These differentials were previously assessed for their response to the majority of the treatments ([25,39,40], TE de Boer, MJTN Timmermans, unpubl. data). For the desiccation and starvation treatments in *O. cincta*, lack of prior knowledge of characteristic responsive genes made us look to a fixed set of two differentials for all four treatments. QPCR assays for seven candidate reference genes (Table 1) and two genes differential for a range of stressful conditions, *HSP70 *and *MT*, [41,42] were developed. For *F. candida*, eight candidate reference genes were analyzed (Table 1) together with one differential gene for each treatment. Primers were based on sequences present in the Collembola EST database Collembase  and generated with Primer Express 1.5 (Applied Biosystems) with the following settings: Melting temperatures were kept between 59°C and 60°C and the amplified fragment length was kept between 90 and 120 base pairs with an optimum amplicon melting temperature of 80°C. For primer sequences, GC content and melting temperatures (see Additional file 4). Details on the positions of the QPCR amplicons in the full coding sequences are given in (Additional file 5).

Reaction efficiency of each of the QPCR assays was determined by means of a standard curve consisting of 5 samples each four fold diluted, from an initial cDNA pool. Each reaction was carried out in a total volume of 20 μL, using 2 μL cDNA template, 10 μL SYBR Green I master mix (Applied Biosystems) and 20 pmole of each gene specific primer (supplied by Isogen). QPCR Cycling was performed on an DNA engine Opticon1 (Biorad), with three technical replicates per sample. Cycling conditions were kept constant for all assays. For details on PCR mix and program see Roelofs et al. [25]). Specificity of the PCR products was confirmed after each amplification by analysis of the melting curve; 60–90°C with a heating rate of 0.1°C per second and one fluorescence measurement per second. Each run included a non-template control for each assay.

For two of the *O. cincta *assays (*GAPDH *and *SDHA*) no sequence information was available. Therefore degenerate primers were developed based on sequences from multiple organisms that were taken from Genbank. Generated PCR templates were subsequently cloned, sequenced and used as a template for QPCR primers as described above. Proper QPCR data were not retrieved from one *F. candida *pH treatment replicate (pH 3.5). Therefore this sample was discarded from further analysis.

### Data analysis

Ct values were calculated with the Opticon Monitor 3 software (Biorad), using a manually set cycle threshold. At a level of 0.01 raw fluorescent units which in all assayed plates fell within the exponential phases of the QPCR reactions. Averages of the three technical replicates were used in case of a standard deviation ≤ 0.5. When standard deviation exceeded this number, fluorescence curves were evaluated. The analysis was always based on at least two replicates.

Ranking of reference genes was determined using geNorm and Normfinder applications, as implemented in the Genex Light software package [43]. Optimum numbers of reference genes are based on a Vn/Vn+1 value of > 0.15 for geNorm [15] and the minimum of accumulated standard deviations for Normfinder [17]. The original geNorm VBA applet for Excel was used for automated calculation of Vn/Vn+1.

Relative gene expressions of all differential genes were calculated with the Pfaffl method [10] and normalized with both of the optimum sets of reference genes proposed by geNorm and Normfinder. Significance levels were tested by Student's t-test, for both algorithms. To be conservative we report only the larger P-value in the results. The temperature exposures of *F. candida *(Figure 2) were normalized as described in the results section, and tested for their significance by one-way ANOVA analysis with a Bonferroni corrected post-hoc test between the different temperatures for each normalization method.

### Bias threshold definition

In Normfinder the total stability ('variability') of a gene is defined by the magnitude of the intragroup variation relative to the intergroup variation [16]. Differential genes, which are responding to one or more treatments, will greatly increase the intergroup variation and hence have a disproportionally large effect on the calculated variability and the ranking order of the other candidate genes. To avoid such biases, differential genes should be excluded from the NormFinder analysis using a pre-selection procedure. The pre-selection procedure consisted of an initial NormFinder analysis with all genes, from which we calculated a bias threshold for the amount of total absolute bias a gene was allowed to have, compared to the total mean absolute bias of the group. Assuming a normal distribution of biases and genes, we considered the 10% most stable genes to be suitable as potential reference genes, which sets the bias threshold between -0.13*SD and 0.13*SD of the normal distribution [44]. In the final NormFinder analysis only those genes that met the criterion of being among the 10% most stable genes were included. Ranking of the remaining genes was subsequently done by determining the standard deviation in a Normfinder analysis with the software settings set to not taking groups into account (M. Kubista, pers. comm.).

## Authors' contributions

MEB, TEB, DR, JE and NMvS conceived the study, set up its design and drafted the manuscript. MEB and TEB conducted the *F. candida *experiments and the QRT-PCR work. JE and DR performed the *O. cincta *treatments and RNA extractions. JM sequenced and designed the QPCR assays for the unknown genes. MJTNT partially generated material for the *F. candida *desiccation analysis and helped with QPCR assay design. BN helped conducting some of the treatments. All authors helped shaping and approved the final manuscript.

## Supplementary Material

Additional file 1 **Expression levels of reference genes and differentially expressed genes in *Folsomia candida *and *Orchesella cincta*.** Click here for file

Additional file 2 **GeNorm analyses of species overlapping treatments and genes.** Click here for file

Additional file 3 **Normfinder analyses of *Orchesella cincta *genes over all treatments.** Click here for file

Additional file 4 **Primer sequences and parameters for the QPCR assays used in this study.** Click here for file

Additional file 5 **Locations of QRT-PCR amplicons in coding sequence.** Click here for file
