# Supplementary material for: Reference genes for QRT-PCR tested under various stress conditions in Folsomia candida and Orchesella cincta (Insecta, Collembola)
Source: BMC Mol Biol. 2009 Jun 1;10:54. doi: 10.1186/1471-2199-10-54 (PMC2698932; doi:10.1186/1471-2199-10-54)
Supplement: Additional file 1 — Expression levels of reference genes and differentially expressed genes in Folsomia candida and Orchesella cincta. [file 1471-2199-10-54-S1.doc]

**Additional file 1 – Expression levels of reference genes and differentially expressed genes in *Folsomia candida* and *Orchesella cincta***

Expression levels of candidate reference genes (*28S*= 28S ribosomal RNA; *ACTb*= beta actin; *CYP*= cyclophilin A; *EF1a*= elongation factor 1α; *ETIF*= eukariotic translation initiation factor 1A; *GAPDH*= glyceraldehyde-3P-dehydrogenase; *SDHA*= succinate dehydrogenase; *UBC*= ubiquitin conjugating enzyme; *TBA*= alpha tubulin; *YWHAZ*= tyrosine 3-monooxygenase) and differentially expressed genes (*HSP70*= heat shock protein 70; *MT*= metallothionein *ATPase*= V-type ATPase *BCS1*= mitochondrial chaperone BCS1; *CP*= cuticle protein) in *Folsomia candida* and *Orchesella cincta* averaged across treatments. Values are given as QRT-PCR cycle threshold values (Ct). Boxes represent the lower and upper quartiles with medians; whiskers illustrate the 10 to 90 percentiles of the samples. Circles represent outliers.


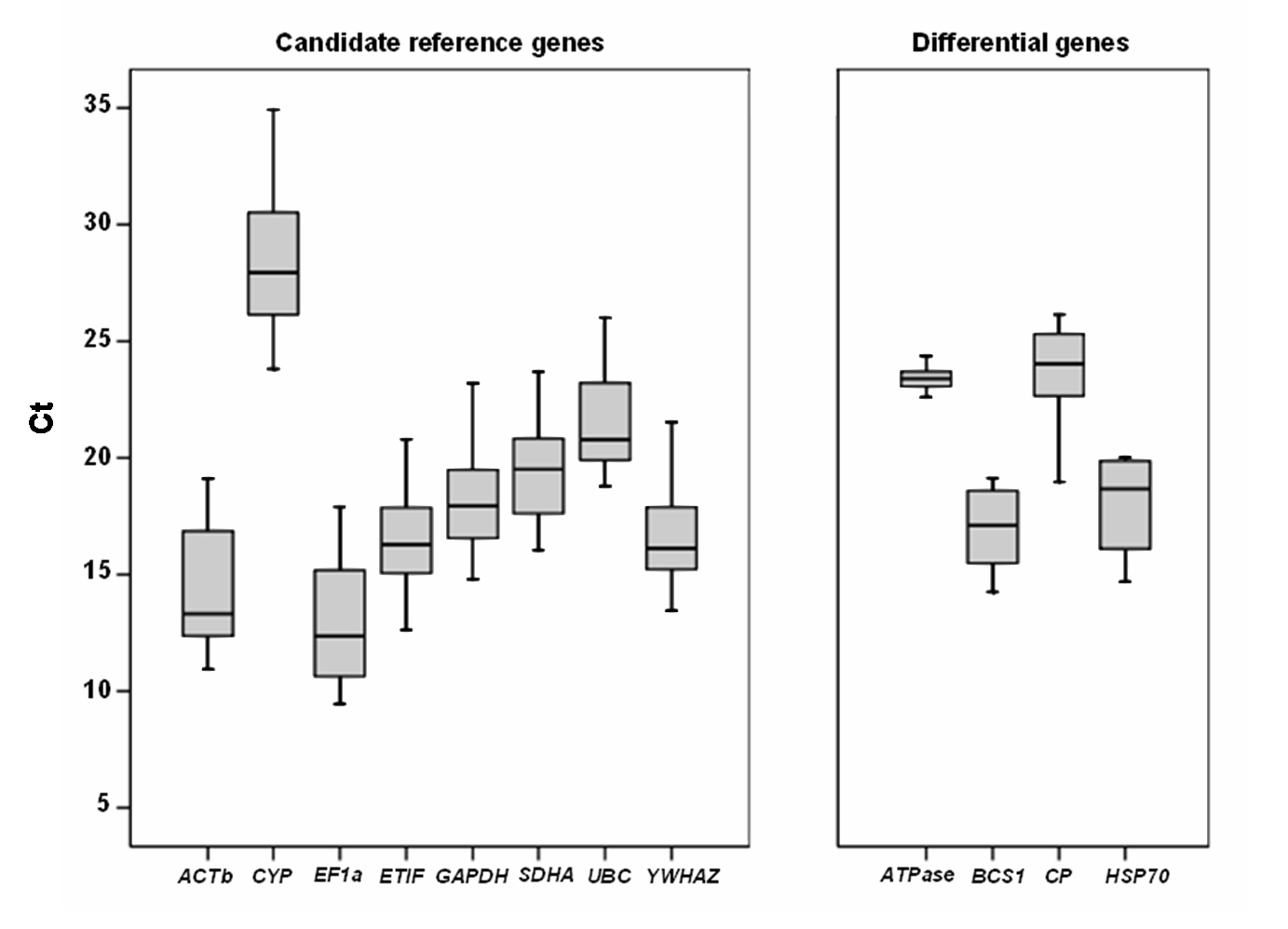

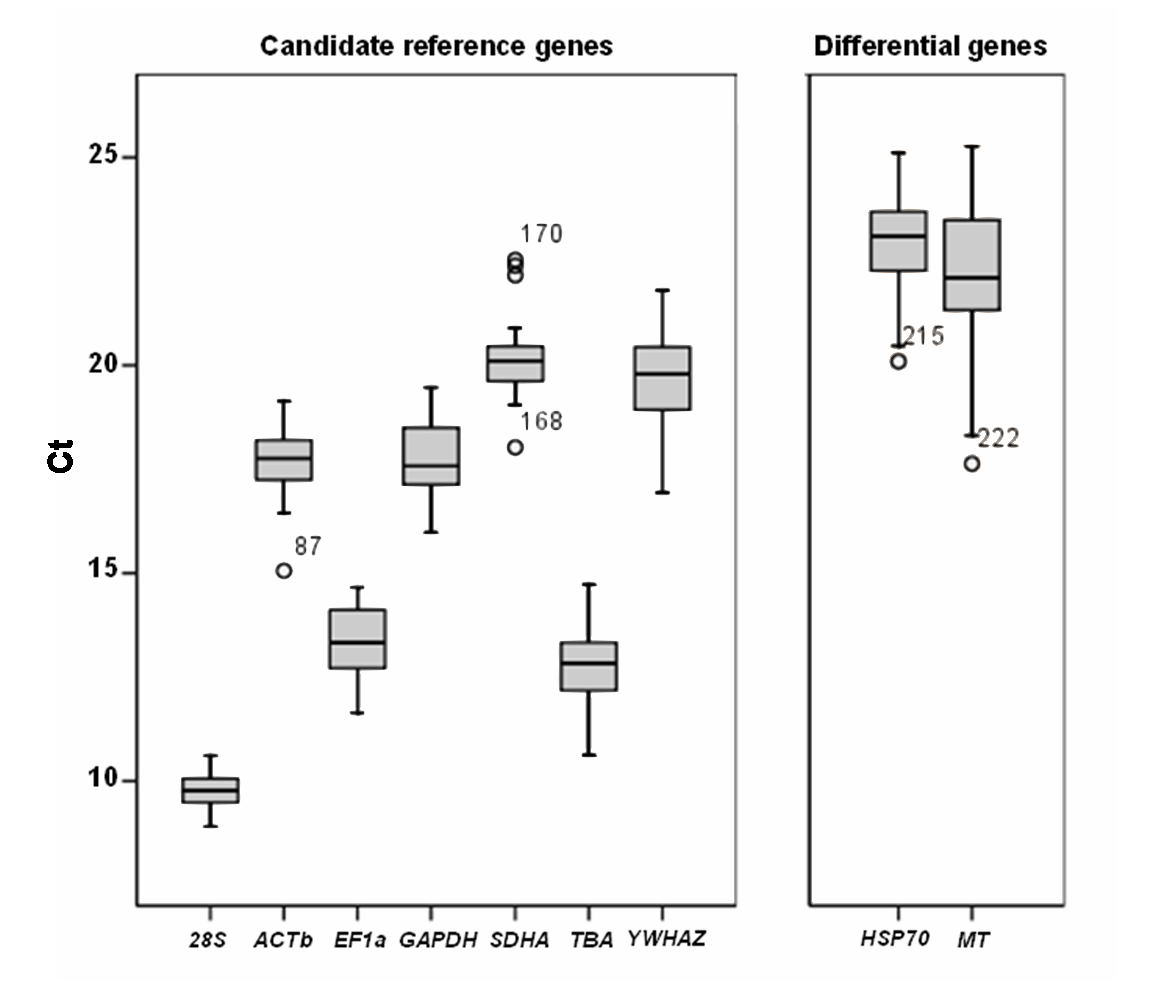

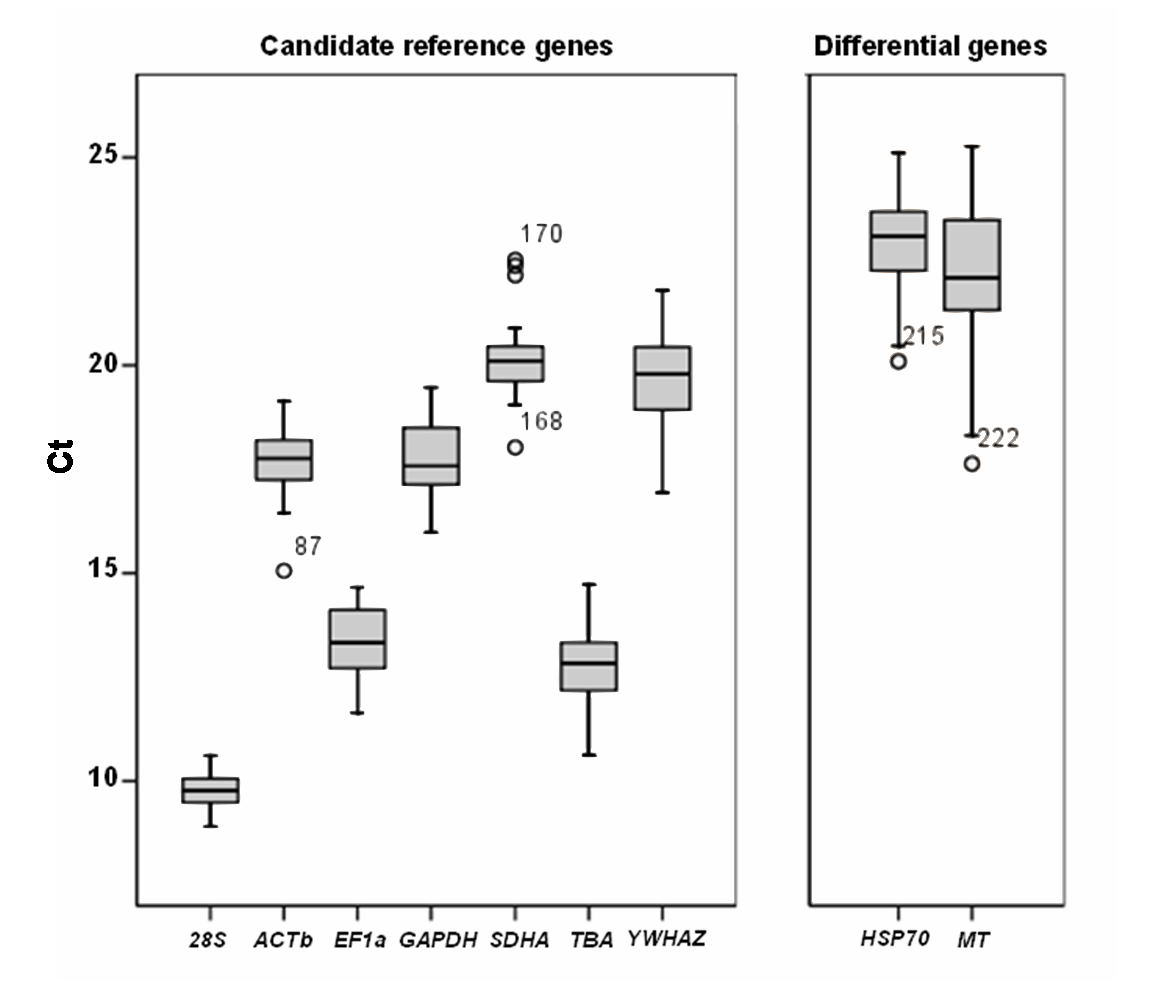


***Folsomia candida***

***Orchesella cincta***
