# Supplementary material for: Reference genes for QRT-PCR tested under various stress conditions in Folsomia candida and Orchesella cincta (Insecta, Collembola)
Source: BMC Mol Biol. 2009 Jun 1;10:54. doi: 10.1186/1471-2199-10-54 (PMC2698932; doi:10.1186/1471-2199-10-54)
Supplement: Additional file 2 — GeNorm analyses of species overlapping treatments and genes. [file 1471-2199-10-54-S2.doc]

temperature

temperature

desiccation

desiccation

cadmium

cadmium

***Folsomia candida***

***Orchesella cincta***

**Additional file 2 – GeNorm analyses of species overlapping treatments and genes**

Temperature, desiccation and cadmium treatments including only candidate reference genes measured for *Folsomia candida* as well as *Orchesella cincta*, analysed by geNorm. Red bars indicate the best pair of selected reference genes. Note that this is a restricted set of housekeeping genes and therefore the rankings can differ from those given in Table 3.
