# Supplementary material for: Reference genes for QRT-PCR tested under various stress conditions in Folsomia candida and Orchesella cincta (Insecta, Collembola)
Source: BMC Mol Biol. 2009 Jun 1;10:54. doi: 10.1186/1471-2199-10-54 (PMC2698932; doi:10.1186/1471-2199-10-54)
Supplement: Additional file 3 — Normfinder analyses of Orchesella cincta genes over all treatments. [file 1471-2199-10-54-S3.doc]

**Additional file 3 – Normfinder analyses of *Orchesella cincta* genes over all treatments**

Outputs of Normfinder analysis for temperature, desiccation, cadmium and starvation treatments of *Orchesella cincta* genes. Left graph shows results for the analysis including all genes and groups set to ‘treatments’. The right graph shows the results for the genes that showed little bias, and Normfinding set to not taking groups into account.
