# Supplementary material for: Reference genes for QRT-PCR tested under various stress conditions in Folsomia candida and Orchesella cincta (Insecta, Collembola)
Source: BMC Mol Biol. 2009 Jun 1;10:54. doi: 10.1186/1471-2199-10-54 (PMC2698932; doi:10.1186/1471-2199-10-54)
Supplement: Additional file 4 — Primer sequences and parameters for the QPCR assays used in this study. [file 1471-2199-10-54-S4.doc]

**Additional file 4 - Primer sequences and parameters for the QPCR assays used in this study**

| **gene** | ***Folsomia candida primers* (5’ → 3’)** | **efficiency** | **Rsq** | ***Orchesella cincta* primers (5’ → 3’)** | **efficiency** | **Rsq** |
| --- | --- | --- | --- | --- | --- | --- |
| *ACTb* | Forward: GAA GTG CGA CGT TGA TAT CCG  Reverse: CAA GGC AGT GAT TTC CTT TTG C | 1.85 | 0.952 | Forward: CCG TAA GGA TCT GTA TGC CAA CA  Reverse: GGC AGT GAT CTC CTT TTG CAT C | 2.05 | 0.998 |
| *GAPDH* | Forward: TTG GAA GGT GGA GCT AAG CGA  Reverse: TCA TCG AAG GAT CAT AGG CGG | 1.95 | 0.996 | Forward: ATG ACC ACT GTT CAT GCT ATC ACT G  ReverseL CAG GAA TGA TAT TTT GTG CAG CAC | 1.91 | 0.999 |
| *UBC* | Forward: CGT TTA CGC AGA TGG TGG AAT C  Reverse: GGA TCG CTC AGA AGT GAC TGG A | 1.93 | 0.997 | Forward: n/a  Reverse: n/a | n/a | n/a |
| *SDHA* | Forward: ACA CTT TCC AGC AAT GCA GGA G  Reverse: TTT TCA GCC TCA AAT CGG CA | 1.99 | 0.995 | Forward: CAA ACA CAC GCT GCT GTA TTC AG  Reverse: CCA CGG TCA AAG AGC TTC AAG T | 1.88 | 0.995 |
| *YWHAZ* | Forward: TCG CCC TCA ACT TTT CCG TT  Reverse: TGC TAT CGC TTC ATC GAA TGC T | 1.89 | 0.999 | Forward: TGA GGC GAT AGC TGA GTT GGA T  Reverse: GTC CAA AGC GTC AAG TTG TCG | 2.13 | 0.995 |
| *EF1a* | Forward: CAC GAT AAG CAC AGC GCA ATC  Reverse: CCG CAA AGT TCT ACG TCA CCA T | 1.88 | 0.995 | Forward: tac tgg tga gtt cga agc tgg tat c  Reverse: CAG TGG AGT CCA TCT TGT TGA CAC | 2.00 | unreported |
| *Etif* | Forward: TGA TTC TGG AGA TCT TCG CGA G  Reverse: ACA GTG CAA AGG ATT TCC CGA | 1.88 | 0.998 | Forward: n/a  Reverse: n/a | n/a | n/a |
| *CYP* | Forward: CAT TCC AAG TAG GTC CCC TTC G  Reverse: CAT CAA TGT CTT CAT CTG CCG C | 2.10 | 0.964 | Forward: n/a  Reverse: n/a | n/a | n/a |
| *28S* | Forward: n/a  Reverse: n/a | n/a | n/a | Forward: TAC GCC GCA TTT GTT CCT CT  Reverse: ACC AGG ACT CAA ACC ACA AGG AT | 2.03 | unreported |
| *TBa* | Forward: n/a  Reverse: n/a | n/a | n/a | Forward: AAG ACG TCA ACG CTG CAA TTG  Reverse: GTT GAT ACC GAC CTT GAA ACC AGT | 1.67 | 0.995 |
| *HSP70* | Forward: TTG GTC GAC GTA GCT CCA CTC T  Reverse: TGG GCT TGT TTG CAT GGA AT | 2.00 | 0.953 | Forward: ATC AAA GTT TAT GAG GGA GAG CGA  Reverse: AAG GTG ACT TCA ATT TGT GGC AC | 1.78 | 0.999 |
| *ATPase* | Forward: CCC GAG TTA AGG AGA TTC TGC A  Reverse: TCC AAC GTG ATT TAT CCC GTC T | 1.98 | 0.970 | Forward: n/a  Reverse: n/a | n/a | n/a |
| *CP* | Forward: AAC ACC CAT AGT CAG CAG GGA A  Reverse CC TCG GGA GAC GTG TAG GAA TA | 1.99 | unreported | Forward: n/a  Reverse: n/a | n/a | n/a |
| *BCS1* | Forward: TTT AGA GGT CTG CTT AAC GCG C  Reverse: TAG GGC AGG ATC CAA TCG TTC | 1.86 | 0.997 | Forward: n/a  Reverse: n/a | n/a | n/a |
| *MT* | Forward: n/a  Reverse: n/a | n/a | n/a | Forward: GGC AAA TCG CCC ACT TGT T  Reverse: CCT TGC AGA CAC AAT CTG GAC C | 2.01 | unreported |
