# Supplementary material for: Reference genes for QRT-PCR tested under various stress conditions in Folsomia candida and Orchesella cincta (Insecta, Collembola)
Source: BMC Mol Biol. 2009 Jun 1;10:54. doi: 10.1186/1471-2199-10-54 (PMC2698932; doi:10.1186/1471-2199-10-54)
Supplement: Additional file 5 — Locations of QRT-PCR amplicons in coding sequence. [file 1471-2199-10-54-S5.doc]

| **Additional file 5 - Locations of QRT-PCR amplicons in coding sequence** | | | | | | | | |
| --- | --- | --- | --- | --- | --- | --- | --- | --- |
| A BlastX search (in Swiss Prot) was performed with the QRT-PCR amplicons | | | | | | | | |
|  |  |  |  |  |  |  | |  |
|  | gene | start | end | total CDS |  |  | |  |
| *F. candida* | ACTb | 708 | 816 | 819 |  |  | |  |
|  | cyclo | 525 | 602 | 1863 |  |  | |  |
|  | EF1a | 207 | 311 | 1227 |  |  | |  |
|  | Etif | 321 | 423 | 480 |  |  | |  |
|  | GAPDH | 327 | 426 | 996 |  |  | |  |
|  | SDHA | 1461 | 1557 | 2001 |  |  | |  |
|  | UBC | 234 | 340 | 456 |  |  | |  |
|  | YWHAZ | 513 | 607 | 732 |  |  | |  |
| *O. cincta* | 28S-D2 | 564 | 658 | 4101 |  |  | |  |
|  | ACTb | 723 | 813 | 819 |  |  | |  |
|  | EF1a | 222 | 336 | 1227 |  |  | |  |
|  | GAPDH | 528 | 642 | 996 |  |  | |  |
|  | SDHA | 1572 | 1674 | 2001 |  |  | |  |
|  | TBA | 981 | 1068 | 1347 |  |  | |  |
|  | YWAZ | 630 | 720 | 732 |  |  | |  |
|  |  |  |  |  |  |  | |  |
|  |  |  |  |  |  |  | |  |
|  |  | start = | start nucleotide QRT-PCR amplicon 5' | | | |  | |
|  |  | end = | end nucleotide QRT-PCR amplicon 3' | | | |  | |
|  |  | total = | total amount of nucleotides in the gene | | | |  | |
|  |  |  |  |  |  |  | |  |
